# Self-adapted clustering of solute atoms into a confined two-dimensional prismatic platelet with an ellipse-like quasi-unit cell

**DOI:** 10.1107/S205225251801415X

**Published:** 2018-10-26

**Authors:** Hongbo Xie, Junyuan Bai, Hucheng Pan, Xueyong Pang, Yuping Ren, Shineng Sun, Liqing Wang, Hong Zhao, Boshu Liu, Gaowu Qin

**Affiliations:** a Key Laboratory for Anisotropy and Texture of Materials (Ministry of Education), School of Materials Science and Engineering, Northeastern University, Shenyang 110819, People’s Republic of China

**Keywords:** magnesium alloys, precipitation, prismatic platelets, quasi-unit-cells, DFT, HAADF-STEM

## Abstract

This paper reports a new structured prismatic platelet, self-assembled by an ellipse-like quasi-unit cell, precipitated in Mg–In–Yb and Mg–In–Ca ternary alloys and aged isothermally at 200°C using aberration-corrected high-angle annular dark-field scanning transmission electron microscopy combined with density functional theory computations. The ordered stacking of solute atoms along the [0001]_α_ direction based on elliptically shaped self-adapted clustering leads to the generation of the quasi-unit cell. The bonding of these ellipse-like quasi-unit-cell rods by the Mg atomic columns along the 〈〉_α_ directions formed a two-dimensional planar structure, which has three variants with a {}_α_ habit plane and full coherence with the α-Mg matrix. This finding is important for understanding the clustering and stacking behaviors of solute atoms in condensed matter, and is expected to guide the future design of novel high-strength Mg alloys strengthened by such high-density prismatic platelets.

## Abstract 2

The ordered stacking of solute atoms based on elliptically shaped self-adapted clustering leads to the generation of the quasi-unit cell. The bonding of these ellipse-like quasi-unit-cell rods by the Mg atomic columns forms a two-dimensional planar structure.

## 1. Introduction   

Solute clustering occurs in solution-treated and quenched alloys during natural or artificial aging processes, including most structural alloys of magnesium (Nie, 2012 ▸; Xie *et al.*, 2018*a* ▸,*b* ▸,*c* ▸, 2019 ▸), aluminium (Ringer & Hono, 2000 ▸; Bourgeois *et al.*, 2013 ▸; Pogatscher *et al.*, 2014 ▸; Chen *et al.*, 2006 ▸), titanium (Lütjering & Weissmann, 1970 ▸; Gysler *et al.*, 1974 ▸), nickel (Oblak *et al.*, 1974 ▸; Yoshida *et al.*, 1986 ▸), copper (Jia *et al.*, 2006 ▸; Zhou *et al.*, 2016 ▸) and some steels (Fisher *et al.*, 1953 ▸; Bonny *et al.*, 2009 ▸). It directly affects the structure, morphology and orientation of the precipitated strengthening phase and thus plays an important role in determining the mechanical properties of these alloys. Icosahedral clustering, where a structure has a coordination number of 12, the highest stacking density and the lowest free energy of the system (Frank, 1952 ▸; Travesset, 2017 ▸), usually occurs in aged ternary or multicomponent alloy systems. Precipitation of high-density lamellar structures developed from initial solute icosahedral clusters is an effective way to enhance the strength of alloys such as the T_1_ phase in Al–Li–Cu systems (Gao *et al.*, 2015 ▸; Deng *et al.*, 2017 ▸), the Ω phase in Al–Cu–Mg–Ag systems (Auld *et al.*, 1966 ▸; Kang *et al.*, 2014 ▸) and the γ′′ phase in Mg–RE–Zn systems (Nuttall *et al.*, 1980 ▸; Xia *et al.*, 2015 ▸; Gu *et al.*, 2018 ▸; Xie *et al.*, 2018*d* ▸). For face-centered cubic (f.c.c.) Al alloys, these confined lamellar structures can precipitate along 12 {111}_fcc_ close-packed planes with an equal probability, and such a crisscrossed precipitate network can effectively strengthen the alloys (Gao *et al.*, 2015 ▸; Deng *et al.*, 2017 ▸; Auld *et al.*, 1966 ▸; Kang *et al.*, 2014 ▸). However, these icosahedral cluster-dominated monolayer structures can only precipitate in one of the {0001}_hcp_ basal planes in hexagonal close packed (h.c.p.) Mg alloys, and such an icosahedral cluster is not an ideal obstacle to the basal glide of dislocations (Nie, 2012 ▸).

In contrast to precipitation on close-packed planes, prismatic precipitates could more effectively contribute to precipitation hardening in h.c.p. Mg alloys (Nie, 2012 ▸), but the precipitation of a high density of monolayer prismatic plates is rarely observed in Mg alloys. Recently, Mendis *et al.* (2011 ▸) reported that one kind of prismatic nano-plate can precipitate in the Mg–0.3Ca alloy with the addition of In, which shed light on the design of novel high-strength Mg alloys. However, the atomic structure of the thin prismatic plates, especially with respect to solute stacking and clustering behaviors, is still unknown, and it is thus difficult to correlate such nano-plates with the mechanical properties of Mg alloys. In summary, after more than eighty years since the discovery of the age-hardening effect in the Al–Cu system (Guinier, 1938 ▸; Preston, 1938 ▸), the question remains as to whether there are other confined monolayer structures that can be formed in aged-alloys beyond the icosahedral clusters, as compared with the traditional close-packed plane precipitation.

In this work, we report a new structured high-density confined prismatic platelet precipitated in the Mg–In–Yb and Mg–In–Ca ternary systems and aged isothermally at 200°C. More importantly, using atomic scale high-angle annular dark-field scanning transmission electron microscopy (HAADF-STEM), we determined that the platelet is self-assembled by a quasi-unit cell based on elliptically shaped and self-adapted clustering of solute atoms, which is totally different from any clustering phenomena discovered to date. A new solute-clustering model has been identified and thus proposed. This finding is expected to guide the future design of novel high-strength Mg alloys strengthened by such high-density prismatic platelets.

## 2. Experimental   

Alloys with nominal compositions of Mg–1.0In–0.35Yb and Mg–1.0In–0.35Ca (in at.%) were prepared by melting pure Mg (99.9wt%), In (99.9wt%), Yb (99.9wt%) and Ca (99.9wt%) in an induction furnace under protection of an argon atmosphere. The molten alloys were stirred and kept at 760°C for 5 min and poured into a steel mold preheated to 300°C. The as-cast samples were solution treated at 480°C for 8 h, followed by water quenching and aging at 200°C for 4 h in an oil-bath furnace. Vickers hardness testing was performed by using a hardness tester (W-W-450SVD) with a loading force of 30 N and dwell time of 15 s. The Mg–In–Yb TEM specimens with a diameter of 3 mm were prepared by twin-jet electro-polishing at −40°C in a mixture solution of 5.3 g lithium chloride, 11.2 g magnesium perchlorate, 500 ml methanol and 100 ml 2-butoxy ethanol, followed by ion milling with a low-energy electron beam; and the Mg–In–Ca TEM specimens with a diameter of 3 mm were directly ion-milled with Gatan 695 (5.0 kV ion-gun energy under a 10° milling angle, followed by 3.0 kV ion-gun energy under a 3.5° milling angle). Lastly, a Gatan SOLARUS (950) Plasma Cleaning System was used to clean the sample surfaces. TEM and STEM observations were then carried out using JEM-ARM200F at an accelerating voltage of 200 kV, equipped with probe, a Cs corrector and cold-field emission gun. The probe convergence is 25 mrad which yields a probe size of less than 0.1 nm, and the camera length was set to 8 cm which yields a collection semi-angle of 48–327 mrad.

First-principles calculations were conducted using *VASP* (Kresse & Furthmüller, 1996 ▸) with *PAW* (Blöchl *et al.*, 1994 ▸; Joubert, 1999 ▸) pseudopotentials. The exchange-correlation potential was described by the generalized gradient approximation functional of PBE (Perdew *et al.*, 1996 ▸). The plane-wave cut-off energy was set at 600 eV. The *k*-point sampling-based gamma-centered Monkhorst–Pack scheme was used for the structural relaxations and the geometry optimization process was performed using a conjugate-gradient algorithm until the final force on each atom was less than 0.01 eV Å^−1^. The formation energy in present calculations, *E*_f_, was calculated from following equation
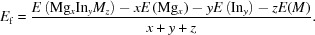



Here, *E*(Mg_*x*_In_*y*_ *M_z_*), *E*(Mg), *E*(In) and *E*(*M*) represent the total energy of the model and the energies of the Mg, In and Yb (Ca) unit cells, respectively.

The present study employed the software *QSTEM* (Setyawan & Curtarolo, 2010 ▸) to simulate HAADF-STEM images, and the corresponding parameters used for simulations are identical with the experimental settings: a voltage of 200 kV, the third-order spherical aberration *C*_3_ = 0.05 mm, defocus value δ*f* = −13.7 nm and the beam convergence angle α = 21.4 mrad.

## 3. Results and discussion   

**Figure 1:**
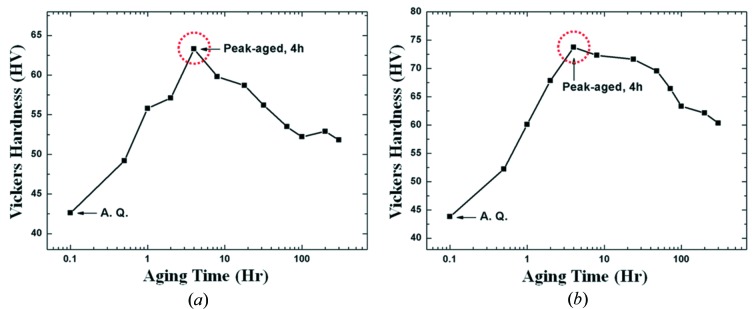
Age-hardening response curves of the (*a*) Mg–In–Yb and (*b*) Mg–In–Ca alloys during isothermal aging at 200°C. These samples were found to have hardness maxima recorded around 4 h, which subsequently decrease with prolonged aging. On the basis of this result, the peak-aged samples (4 h) were selected for TEM and HAADF-STEM characterization.

When the Mg–1.0In–0.35Yb and Mg–1.0In–0.35Ca alloys were solution treated at 480°C for 8 h, followed by water quenching and aging at 200°C for different times, their hardness values changed with aging time as shown in Fig. 1 ▸. The hardness of the Mg–In–Yb alloy increases from ∼42.6 HV in the initial solution-treated state to the highest value of ∼63.3 HV at the peak aging time of 4 h, and then starts to decrease with prolonged aging time. Similarly, the Mg–In–Ca samples were also found to have a hardness maximum recorded around 4 h, the hardness of the alloys increased from 43.8 to 73.7 HV, and subsequently decreased with prolonged aging. The peak-aged sample with the maximum hardness was selected for the following TEM and HAADF-STEM characterization.

**Figure 2:**
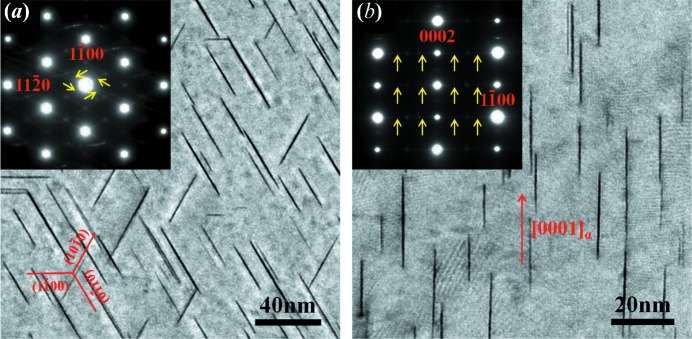
Bright-field TEM images and corresponding SAED patterns of the Mg–In–Yb alloy isothermally aged at 200°C for 4 h. The electron beam is parallel to (*a*) [0001]_α_ and (*b*) [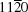]_α_. The insets are the corresponding SAED patterns.

The TEM bright-field images and corresponding selected-area electron diffraction (SAED) patterns of the Mg–In–Yb peak-aged sample, viewed along the [0001]_α_ and [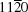]_α_ directions, are shown in Figs. 2 ▸(*a*) and 2(*b*), respectively. As shown in Fig. 2 ▸(*a*), there are high-density platelets with three variants formed along the prismatic {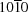}_α_ planes, whose lengths are approximately 20–50 nm, with a thickness <1 nm. The TEM image recorded along the [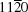]_α_ direction also indicates that the platelets with their habit plane are parallel to the prismatic {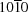}_α_ planes, and are ∼10–30 nm in height (Fig. 2 ▸ *b*). The SAED patterns (shown as insets) exhibit continuous diffraction streaks between the (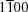)_α_ diffraction spots [yellow arrows in Figs. 2 ▸(*a*) and 2(*b*)], indicating the existence of periodic stacking platelets in the 〈
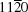
〉_α_ direction.

**Figure 3:**
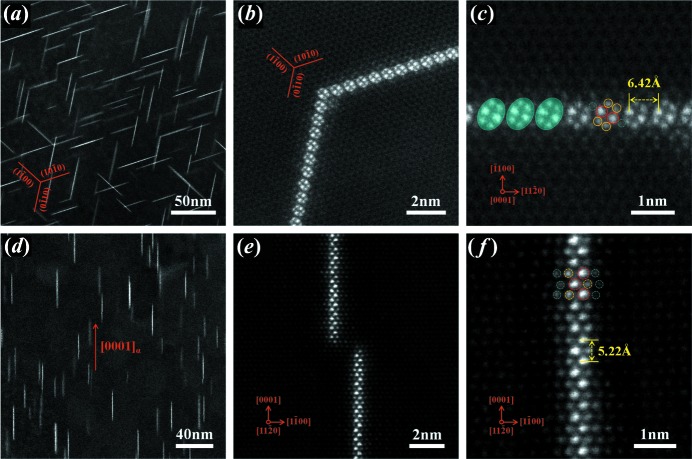
HAADF-STEM images of the Mg–In–Yb alloy isothermally aged at 200°C for 4 h. The electron beam is parallel to (*a*)–(*c*) [0001]_α_ and (*d*)–(*f*) [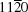]_α_. (*a*) Low-magnification HAADF-STEM image along the [0001]_α_ direction, showing that the bright-contrast platelets have three variants with a {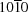}_α_ habit plane. (*b*) and (*c*) An enlarged image of two platelets connected at a 120° angle; the atomic scale HAADF-STEM images show that the structure in the observed two-dimensional image consists of many separate quasi-unit cells with ellipse-like shapes. (*d*) Low-magnification HAADF-STEM image along the [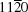]_α_ direction. (*e*) and (*f*) An enlarged image of the platelets in Fig. 3 ▸(*d*); the atomic-scale HAADF-STEM images indicate that the solute atoms are selectively stacked along [0001]_α_.

Fig. 3 ▸(*a*) provides a low-magnification HAADF-STEM image from the [0001]_α_ direction showing the precipitated platelets with bright contrast in the α-Mg matrix, which is consistent with the BF-TEM image shown in Fig. 2 ▸(*a*). To reveal the crystal structure and atomic coordinates of the platelets, atomic imaging by HAADF-STEM was used. Atomic scale HAADF-STEM images of these platelets are enlarged and shown in Figs.  ▸3(*b*) and 3(*c*). These prismatic platelets consist of many ellipse-like solute cluster structures. Two kinds of bright dots with different contrasts inside the ellipse-like structure can be identified: the two brightest dots with large atomic radii located in the center correspond to the Yb atomic columns and the four bright dots with small atomic radii distributed on both sides correspond to the In atomic columns, since the brightness of individual atomic columns in HAADF-STEM images approximates to the square of the average atomic numbers (the atomic numbers are 12 for Mg, 49 for In and 70 for Yb) (Kirkland *et al.*, 1987 ▸; Bos *et al.*, 2016 ▸), as represented by the red and yellow circles in Fig. 3 ▸(*c*), respectively. These ellipse-like structures precipitated along six 〈
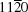
〉_α_ prism planes with an equal probability of generating the present confined platelet, and one new two-dimensional planar structure has three variants with a {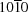}_α_ habit plane. Two platelets connected at a 120° angle were recorded and are shown in Fig. 3 ▸(*b*). Careful analysis of the HAADF-STEM images (Fig. 3 ▸ *c*) indicates that the distance between two ellipse-like structures is ∼6.42 Å. Additionally, a unique feature of the platelet structure, *i.e.* ellipse-like rods, are traced by cyan translucent oval frames in Fig. 3 ▸(*c*).

A low-magnification HAADF-STEM image viewed along [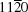]_α_ is presented in Fig. 3 ▸(*d*), and some local parts of the {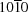} platelets in Fig. 3 ▸(*d*) are further enlarged and shown in Figs. 3(*e*) and 3(*f*). The atomic scale HAADF-STEM images show that the brightest Yb-rich columns and bright In-rich columns exhibit periodic stacking along the [0001]_α_ zone axis. In addition, the interatomic distances of *h*_Yb–Yb_ and *h*_In–In_ were found to be ∼5.22 Å, consistent with the *c* value of the α-Mg matrix.

**Figure 4:**
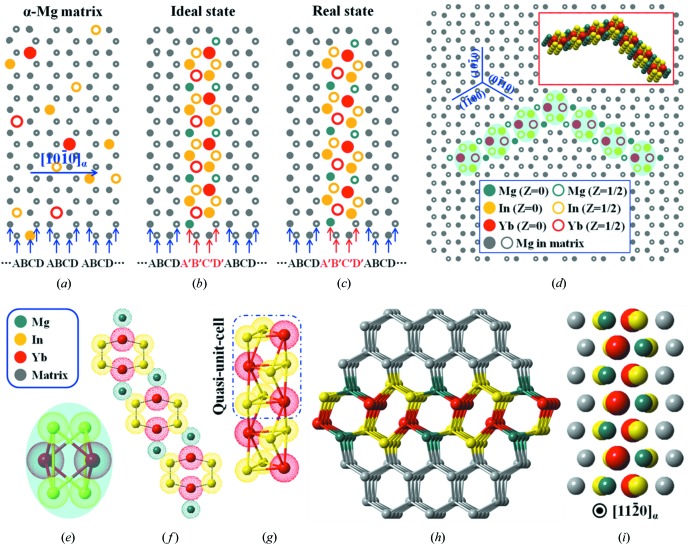
Schematic illustration of the confined two-dimensional prismatic platelet. (*a*) Modeled atomic arrangement of the h.c.p. α-Mg matrix. (*b*) Modeled atomic arrangement of an ideal state for the platelet without considering lattice distortion. (*c*) Modeled atomic arrangement of the real state for the platelet. (*d*) Modeled atomic arrangement for two platelets connected with a 120° angle in the matrix; the top-right inset is the corresponding three-dimensional view. The atomic arrangements (*a*)–(*d*) viewed along the [0001]_α_ direction. (*e*) Atomic structure of the precipitate chain. (*f*) Quasi-unit cell of the platelet. (*g*) Three-dimensional view of the quasi-unit-cell rod. (*h*) Three-dimensional detail of the interface structure between the matrix and platelet. (*i*) Modeled atomic arrangement of the platelet in the matrix viewed along the [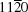]_α_ direction.

A schematic illustration of the prismatic platelet structure is shown in Fig. 4 ▸. Fig. 4 ▸(*a*) shows a modeled atomic arrangement of the supersaturated Mg–In–Yb solid solution viewed along the [0001]_α_ direction, where some Mg positions in the matrix are replaced by solute atoms. During isothermal aging at 200°C, because the affinity between the In and Yb elements is the strongest (the enthalpy of mixing values are −34 kJ mol^−1^ for In–Yb, −4 kJ mol^−1^ for Mg–In and −6 kJ mol^−1^ for Mg–Yb) (Takeuchi & Inoue, 2005 ▸), the In–Yb co-clusters are nucleated preferentially *via* solute diffusion, forming an equilateral hexagonal solute cluster structure perfectly consistent with the matrix lattices, if the atomic size and lattice-distortion factors are not considered (as shown in Fig. 4 ▸ *b*). However, the atomic radii of Yb (1.94 Å) and In (1.66 Å) are both larger than that of Mg (1.60 Å) thus, the formation of In–Yb co-clusters would cause a large strain field near the structure. To reduce strain, the solute atoms self-adapt to clustering to generate the present ellipse-like structure, which has a lower free energy (as shown in Fig. 4 ▸ *c*). From these images, it can be seen that the prismatic planar structure induced by a variation in chemical composition has a stacking sequence of …ABCDA′B′C′D′ABCD… with an …ABCD… sequence of the α-Mg matrix along the [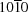]_α_ direction. Fig. 4 ▸(*d*) displays a modeled atomic arrangement for two platelets connected at 120° in the matrix, and the top-right inset is the corresponding three-dimensional view.

Fig. 4 ▸(*e*) shows the atomic structure details of the precipitate chain, where these ellipse-like solute cluster structures are actually bonded together by the Mg atoms. The solute cluster structure, *i.e.* the quasi-unit cell of the platelet, is traced by black hexagonal frames in Fig. 4 ▸(*e*). Fig. 4 ▸(*f*) represents a separate quasi-unit cell, and the corresponding three-dimensional view is shown in Fig. 4 ▸(*g*). From these images, it can be concluded that the prismatic platelet has a stacking sequence of ‘…ABAB…’ along the *z* axis, consistent with the α-Mg matrix. Because of its unique structure, the confined platelet cannot be described simply by the repetition of a single atom or atomic cluster. However, the quasi-unit cell [within the blue dotted box in Fig. 4 ▸(*g*)] can represent the structural characteristic for this platelet along the *z* axis although it does not involve crystallographic symmetry. Fig. 4 ▸(*h*) depicts the three-dimensional detail of the interface structure between the matrix and platelet; the self-adapting distorted platelet structure is fully coherent with the α-Mg matrix. The modeled atomic arrangement viewed along the [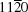]_α_ direction is shown in Fig. 4 ▸(*i*), and it can be determined that each bright dot recorded in the HAADF-STEM images in the [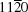]_α_ direction represents a mixed-element column, including the brightest Yb–In columns and brighter In–Mg columns.

The elements Ca and Yb have similar chemical properties; melting points (Ca is 839°C and Yb is 824°C), atomic radii (Ca is 1.98 Å and Yb is 1.94 Å) and electronegativity *etc*. are very close, thus, the valence-fluctuation of Yb is commonly obtained from Ca reduction of Yb_2_O_3_. The enthalpy of mixing values for Ca–In, Mg–In and Mg–Ca are −35, −4 and −6 kJ mol^−1^, respectively (Takeuchi & Inoue, 2005 ▸), which are very close to the values for In–Yb, Mg–In and Mg–Yb. Therefore, the aging precipitation behavior of Mg–In–Yb and Mg–In–Ca systems is almost the same. In a previous study, Mendis *et al.* (2011 ▸) reported that a high number density of {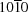}_α_ prismatic platelets can be precipitated in Mg–In–Ca ternary alloys, resulting in an enhanced age-hardening response three times higher than that of the Mg–Ca binary alloy without In (Mendis *et al.*, 2011 ▸). However, the crystal structure and atomic coordinates of the {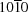}_α_ prismatic platelet are still unknown at present.

**Figure 5:**
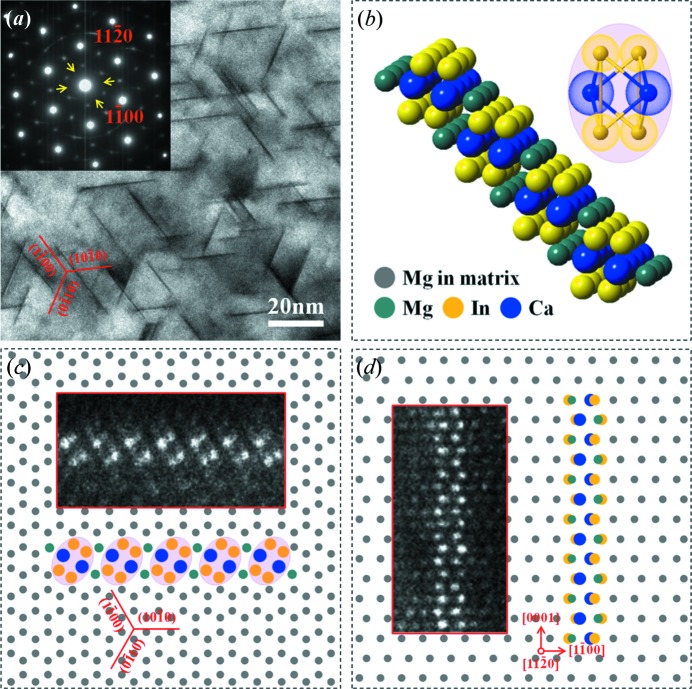
(*a*) Bright-field TEM image and corresponding SAED patterns of the Mg–In–Ca alloy isothermally aged at 200°C for 4 h. The electron beam is parallel to [0001]_α_. (*b*) Atomic structure of the Mg–In–Ca prismatic platelet. (*c*) and (*d*) Modeled atomic arrangement of the Mg–In–Ca prismatic platelet, viewed along (*c*) [0001]_α_ and (*d*) [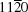]_α_, the insets are the corresponding atomic scale HAADF-STEM images.

Fig. 5 ▸(*a*) provides a [0001]_α_ TEM bright-field image of the Mg–In–Ca alloy isothermally aged at 200°C for 4 h, and the top-left inset is the corresponding SAED pattern. It can be seen that the precipitated platelets with three variants are formed along the prismatic {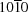}_α_ planes, which is similar to the precipitation behavior of the Mg–In–Yb alloy, as presented in Fig. 2 ▸(*a*). Atomic scale HAADF-STEM images for the platelet, viewed along the [0001]_α_ and [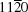]_α_ directions are shown as insets in Figs. 5(*c*) and 5(*d*), respectively. It can be shown that there are significant differences between the two kinds of {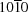}_α_ prismatic platelets under HAADF-STEM imaging, and the positions of Yb with the brightest contrast in the Mg–In–Yb platelet are almost invisible in the Mg–In–Ca platelet, since the atomic numbers of Ca and Mg are close to each other (the atomic numbers are 12 for Mg, and 20 for Ca) (Kirkland *et al.*, 1987 ▸; Bos *et al.*, 2016 ▸). The modeled atomic arrangements of the Mg–In–Ca prismatic platelet viewed along the [0001]_α_ and [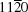]_α_ directions are shown in Figs. 5(*c*) and 5(*d*), and the corresponding three-dimensional view is presented in Fig. 5 ▸(*b*). Similarly with the Mg–In–Yb platelet, the solute atom (Ca and In elements) ordered stacking along the [0001]_α_ direction based on elliptically shaped self-adapted clustering leads to the generation of the quasi-unit cell. The bonding of these ellipse-like quasi-unit cell rods by the Mg atomic columns along 〈
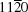
〉_α_ generates the two-dimensional planar structure presented here.

**Figure 6:**
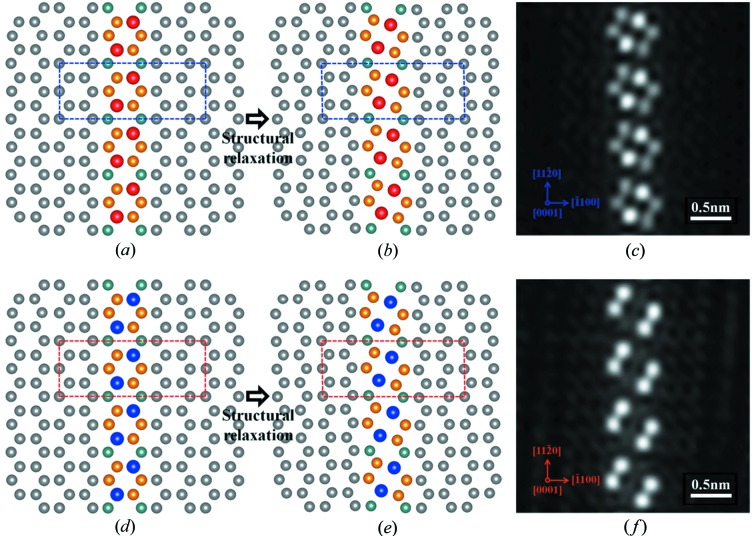
DFT computations and HAADF-STEM simulation results viewed along [0001]_α_. The atomic arrangements of the Mg–In–Yb and Mg–In–Ca prismatic platelets at (*a*) and (*d*) ideal and (*b*) and (*e*) relaxed states in the simulation processes. Corresponding simulated HAADF-STEM images of the (*c*) Mg–In–Yb and (*f*) Mg–In–Ca prismatic platelets.

In order to verify the validity and rationality of the proposed structure, first-principles calculations have been performed. The unrelaxed structures of the Mg–In–Yb and Mg–In–Ca prismatic platelets, *i.e.* the ideal states without considering lattice distortion and rearrangements of atoms, are shown in Figs. 6(*a*) and 6(*d*), and the corresponding geometry relaxed platelet structures are presented in Figs. 6(*b*) and 6(*e*). It is obvious that the optimized structures are different from the ideal states, and the equilateral hexagonal solute clusters transit to the ellipse-like solute clusters *via* self-adapted shuffling. The corresponding simulated HAADF-STEM images for the relaxed structures are shown in Figs. 6(*c*) and 6(*f*), respectively, which are consistent with the experimental HAADF-STEM observations shown in Figs. 3(*c*) and 5(*c*), indicating that the two-dimensional prismatic platelets with an ellipse-like quasi-unit cell can be generated and exist in a stable state. The formation energies *E*_f_ of two prismatic platelets are −81.60 meV for Mg–In–Yb and −79.07 meV for Mg–In–Ca, indicating that the optimized structures are thermodynamically stable, and further proves that our proposed structures are theoretically correct and rational.

Compared with the basal precipitation behaviors, such as the Laves phase (Xie *et al.*, 2018*a* ▸), γ′′ phase (Nuttall *et al.*, 1980 ▸; Xia *et al.*, 2015 ▸; Gu *et al.*, 2018 ▸; Xie *et al.*, 2018*b* ▸), or long-period-stacking order phase (Yamasaki *et al.*, 2007 ▸; Iikubo *et al.*, 2012 ▸) in Mg alloys, the platelets precipitated in the prism planes usually exhibit a stronger strengthening effect (Nie, 2012 ▸). In this work, the Mg–In–Yb alloy shows an aging-hardening maximum hardness of 63.3 HV, an ∼50% increase. Also the Mg–In–Ca alloy performs better and the hardness is increased by ∼68% (Fig. S1*b*). If a higher precipitation density can be designed, or the prismatic platelets and the basal one can be precipitated simultaneously to form a crisscrossed precipitation network, even higher strength for the Mg alloy would be expected. However, the hardness subsequently decreases with prolonged aging. One reason for this result is that the growth and coarsening of these nano-platelets lead to a decrease in precipitate density. As shown in Fig. S1(*b*), the bright-field TEM image indicates that the coarsening Mg–In–Ca platelets still exist stably in the matrix after isothermal aging at 200°C for 100 h. For the Mg–In–Yb system however, more crucially these platelets transform into a coarsening unknown phase with prolonged aging, as shown by the bright-field TEM image shown in Fig. S1(*a*), where three ‘S’-shaped variants, like carpenterworms, are distributed along the {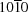}_α_ planes (200°C, 100 h). In other words, the present nano-scale platelet structure found in the Mg–In–Yb alloy is a metastable cluster.

## 4. Conclusions   

We have discovered high-density prismatic platelets precipitated in Mg–In–Yb and Mg–In–Ca ternary alloys aged isothermally at 200°C. Based on atomic scale HAADF-STEM observations and DFT computation, the crystal structures and atomic coordinates of the prismatic platelets are fully revealed. Self-adapted clustering of the solute atoms, in selected stacking along [0001]_α_, generates an ellipse-like quasi-unit cell. The bonding together of these quasi-unit cells by Mg atoms along the 〈
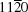
〉_α_ directions leads to the generation of a platelet with a new two-dimensional planar structure, fully coherent with the matrix, and with three variants with the {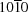}_α_ habit plane. A new solute clustering model has thus been confirmed. The findings presented here not only provide practical theoretical guidance for designing and developing novel high-strength Mg alloys *via* prismatic platelet strengthening, but also hope to aid future understanding of the clustering and stacking behaviors of solute atoms in condensed matter.

## Supplementary Material

Supplementary TEM images. DOI: 10.1107/S205225251801415X/zx5014sup1.pdf
