# Supplementary material for: Self-adapted clustering of solute atoms into a confined two-dimensional prismatic platelet with an ellipse-like quasi-unit cell
Source: IUCrJ. 2018 Oct 26;5(Pt 6):823–9. doi: 10.1107/S205225251801415X (PMC6211520; doi:10.1107/S205225251801415X)
Supplement: Supplementary file 1 [file m-05-00823-sup1.pdf]

IUCrJ

Volume 6 (2019)

Supporting information for article:

Self-Adapted Clustering of Solute Atoms into a Confined Two-Dimensional Prismatic Platelet with an Ellipse-like Quasi-Unit-Cell

Hongbo Xie, Junyuan Bai, Hucheng Pan, Xueyong Pang, Yuping Ren, Shineng Sun, Liqing Wang, Hong Zhao, Boshu Liu and Gaowu Qin

Self-Adapted Clustering of Solute Atoms into a Confined Two-Dimensional Prismatic Platelet with an Ellipse-like Quasi-Unit-Cell

Supporting information

Hongbo Xie†, Junyuan Bai†, Hucheng Pan, Xueyong Pang, Yuping Ren*, Shineng Sun, Liqing Wang, Hong Zhao, Boshu Liu, Gaowu Qin*

Key Laboratory for Anisotropy and Texture of Materials (Ministry of Education), School of Materials Science and Engineering, Northeastern University, Shenyang 110819, China

† These authors contributed equally to this work.

* Corresponding author. Email: renyp@atm.neu.edu.cn (Y. P. Ren); qingw@smm.neu.edu.cn (G. W. Qin).

Supplementary Figures

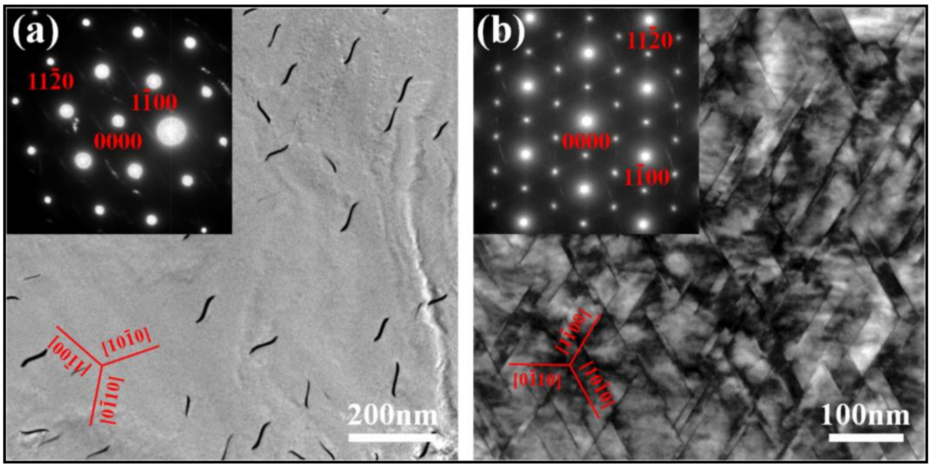

Figure S1 Bright-field TEM images and corresponding selected-area electron diffraction (SAED) patterns of the Mg-In-Yb and Mg-In-Ca alloys isothermally aged at 200 ºC for 100 h, The electron beam is parallel to $[0001]_{\alpha}$. (a) Bright-field TEM image showing the “S”-shaped unknown phase transformed from the 2D platelet structure in the Mg-In-Yb system. (b) Bright-field TEM image indicated that the prismatic platelet structure can stably exist in the Mg-In-Ca system.
